# Small-cell lung cancer-associated autoantibodies: potential applications to cancer diagnosis, early detection, and therapy

**DOI:** 10.1186/1476-4598-10-33

**Published:** 2011-03-30

**Authors:** Meleeneh Kazarian, Ite A Laird-Offringa

**Affiliations:** 1 Departments of Surgery and of Biochemistry and Molecular Biology, Norris Cancer Center, Keck School of Medicine, University of Southern California, 1441 Eastlake Ave. NOR 6420, Los Angeles, CA 90089-9176, USA

## Abstract

Small-cell lung cancer (SCLC) is the most aggressive lung cancer subtype and lacks effective early detection methods and therapies. A number of rare paraneoplastic neurologic autoimmune diseases are strongly associated with SCLC. Most patients with such paraneoplastic syndromes harbor high titers of antibodies against neuronal proteins that are abnormally expressed in SCLC tumors. These autoantibodies may cross-react with the nervous system, possibly contributing to autoimmune disease development. Importantly, similar antibodies are present in many SCLC patients without autoimmune disease, albeit at lower titers. The timing of autoantibody development relative to cancer and the nature of the immune trigger remain to be elucidated. Here we review what is currently known about SCLC-associated autoantibodies, and describe a recently developed mouse model system of SCLC that appears to lend itself well to the study of the SCLC-associated immune response. We also discuss potential clinical applications for these autoantibodies, such as SCLC diagnosis, early detection, and therapy.

## Introduction

Lung cancer is the leading cause of cancer-related death in the world, claiming the lives of 1.3 million individuals worldwide in 2007 [1]. In the United States, lung cancer killed over 150,000 Americans in 2009 [2,3] and caused more deaths than breast, prostate, pancreatic, and colon cancer combined. Small-cell lung cancer (SCLC), a highly malignant tumor thought to originate from primitive neuroendocrine cells in the lung [4], accounts for up to 15% of all newly diagnosed lung cancers [5]. Cigarette smoking is the major cause of SCLC, where both the smoking intensity (cigarettes/day) and the number of years of smoking increase the risk of SCLC development [6]. Recently, it was shown that repetitive nicotine exposure induces many malignant features in SCLC cells, including increased adhesion, enhanced migration, and resistance to chemotherapy [7]. Initially, SCLC patients respond well to chemotherapy. However, relapses are inevitable as patients become resistant to cytotoxic treatment [8]. Despite treatment, the relative 5-year survival is only 6.4% [3], making SCLC the most aggressive lung cancer subtype.

There are as yet no effective early detection tools for SCLC. It is most often diagnosed due to symptoms associated with disseminated disease, such as bulky intrathoracic malignancy or distant metastases. Cough, shortness of breath, and chest pain are the most common local symptoms, and distant signs of the disease include weight loss and weakness. After presentation of symptoms, histological analysis of bronchoscopic biopsy samples and cytological study of fine-needle aspiration (FNA), transbronchoscopic needle aspiration (TBNA), or endoscopic ultrasound (EUS)-guided fine-needle aspiration (EUS-FNA) samples are common approaches to confirm SCLC diagnosis [9-12]. The cancer is defined as a malignant epithelial tumor consisting of small cells with altered cytoplasm, ill-defined cell borders, granular nuclear chromatin, and absent or inconspicuous nucleoli. The cells can be round, oval, or spindle-shaped [13]. It can be difficult to pathologically distinguish SCLC from other lung malignancies, including neuroblastoma, embryonal rhabdomyosarcoma, desmoplastic small round cell tumor, primitive peripheral neuroectodermal tumors [14], poorly differentiated squamous cell carcinomas, and large cell carcinomas [10]. Epithelial markers, such as cytokeratins, and neuroendocrine markers can be employed to differentiate SCLC tumors from the aforementioned lung malignancies. Sampling error is the most commonly reported cause of false negatives in lung FNA cytology [10,15]. SCLCs are centrally located, and accessing them by FNA is more difficult in comparison to peripherally located adenocarcinomas and metastatic neoplasms [10]. In addition, the small size of the cells increases the chances of crushing the sample by biopsy forceps or distorting the sample during needle aspiration [16]. Given that accurately diagnosing SCLC can be difficult, the development of additional methods, such as detection of molecular markers associated with this disease, may increase the efficacy of diagnosis. Molecules that might be suitable for this purpose are SCLC-associated autoantibodies. Examples of such antibodies are those found in paraneoplastic neurologic syndromes associated with SCLC.

Paraneoplastic neurologic syndromes (PNS) are defined as cancer-associated neurological diseases that damage neuronal tissues in a site remote from the tumor (unrelated to metastasis) [17]. PNS patients typically harbor antibodies directed against neuronal antigens that are abnormally expressed in the tumor. Thus, the tumor and the immune system are both implicated in the development of PNS [18-20]. A number of PNS are strongly associated with SCLC. They are severely debilitating and often are the cause of death in SCLC patients who are affected by them. While these autoimmune diseases are quite rare, affecting a small percentage of SCLC patients [18], the characteristic antibodies can actually be found in a substantial fraction of SCLC patients without neurological symptoms, albeit at low titers. This suggests that these antibodies may have utility for early detection and diagnosis of SCLC. In order to be of use for early detection, the timing of the antibody response in relation to that of SCLC development and progression has to be clearly established. In the case of SCLC-associated PNS, the diagnosis of the neurological disease often antedates that of the tumor [18,21], suggesting that the body's immune system can detect the presence of SCLC before the cancer becomes symptomatic. If such antibodies were to generally arise prior to tumor metastasis in SCLC patients, they could potentially provide an avenue for early detection.

The mechanism by which autoimmunity develops in SCLC remains to be elucidated. For example, it is unclear whether the antigens that prompt an immune response are in any way different from those in normal tissue. If these antigens exhibited cancer-specific changes responsible for triggering an antibody response, they might not only be useful for the development of tools for (early) detection, but also for imaging and treatment. Thus, elucidating the basis of immune reactivity in SCLC is of great importance. The study of SCLC-associated PNS offers a potential window into the relationship between SCLC and the immune response.

## SCLC-related autoantigens and autoantibodies

### Overview of paraneoplastic syndromes associated with SCLC

Many SCLC-associated autoantigens are normally expressed in the nervous system, but they become abnormally expressed in the tumor, classifying them as onconeural antigens. The biological characteristics of these autoantigens can be broadly divided into four categories: 1) neuromuscular junction proteins; 2) vesicle-associated nerve-terminal proteins; 3) neuron-specific DNA or RNA-binding proteins; and 4) neuronal signaling proteins [22]. Several different onconeural antigens can give rise to the same disease phenotype, as seen in Table 1. Presumably this is because these different antigens are normally expressed in the same cells, and the autoimmune response attacks the same parts of the nervous system. The mechanism by which the immune system identifies these antigens as foreign remains poorly understood. Importantly, the immune response includes the generation of autoantibodies, which have been crucial tools in gaining an understanding of the cause and pathology of these cancer-associated syndromes.

**Table 1:** Comprehensive list of SCLC-associated autoantigens and autoantibodies

Autoantigen	Antibody	Paraneoplastic Symptoms	% of antibody- positive PNS patients who have SCLC	% SCLC patients without PNS who have antibodies	Reference
	"Well characterized" onconeural autoantibodies [56]				

Amphiphysin	Anti-amphiphysin	PEM/SN, SMS	2.9	1.4	[27,29]

Collapsin response- mediator protein (CRMP5/POP66)	Anti-CRMP/Anti-CV2	PEM/SN	77	9	[29,142,183-185]

HuB(Hel-N1)/ELAVL2, HuC/ELAVL3, HuD/ELAVL4	Anti-Hu	PEM/SN	85	16-25	[36,42,88,173,186]

Neuro-oncological ventral antigen (Nova)	Anti-Ri (ANNA2)/anti-Nova	PNS; POMA	0	4.5	[29,105,187,188]

Paraneoplastic Ma proteins (PNMA1)	Anti-Ma	PEM/SN	ND	ND	[189,190]

PNMA2	Anti-Ta	PEM/SN	ND	ND	[190]

Surface-binding autoantibodies					

P/Q type voltage-gated calcium channel (VGCC), MysB	Anti-VGCC	LEMS; PCD	40-98	2-5	[50,66,73,74,76,163,164,191,192]

Voltage-gated potassium channel (VGKC)	Anti-VGKC	LEMS	ND	ND	[166,193]

α-amino-3- hydroxy-5-methyl-4-isoxazole-propionic acid receptor (AMPAR)	Anti-AMPAR; Anti-GluR1/2	LEMS	ND	ND	[194]

Protein tyrosine phosphatase receptor type N (PTPRN)	Anti-IA-2	LEMS	25	10	[195]

GABA_B _receptor	Anti-GABA_B_	LEMS	33	ND	[196]

Nicotinic acetylcholine receptors (AChRs)	Anti-AChR (α3)	Autonomic neuropathy	3-5	ND	[197]

Other autoantibodies described in literature and/or case reports					

280-kDa cerebellar protein	Anti-Purkinje cell cytoplasmic antibody (PCA)-2	LEMS	53	2	[29,198]

170-kDa brain protein	Anti-neuronal nuclear autoantibody type 3 (ANNA-3)	LEMS	45	ND	[199]

BR serine/threonine kinase (BRSK)2	Anti-BRSK2	PNS	ND	4	[200]

α-Enolase	Anti-enolase	CAR	ND	0	[201,202]

Glutamic acid decarboxylase (GAD) 2	Anti-GAD65 (GAD65A)	LEMS	63	9-19	[195,203]

Recoverin	Antiretinal, Anti-Rc	CAR	45	10-15	[83,84,87,201,204]

Sry-like high mobility group box (SOX)1,2	Anti-SOX	LEMS	64	22-36	[30,31,78,80,205]

Synaptotagmin (SYT)1	Anti-SYT	LEMS	5	ND	[206]

Synaptophysin	Anti-synaptophysin	PNS	ND	5	[104]

ZIC2 ZIC4 (derived from zinc fingers of cerebellum)	Anti-ZIC	PND	29	16	[80,207,208]

Paraneoplastic encephalomyelitis sensory neuronopathy (PEM/SN); Lambert-Eaton myasthenic syndrome (LEMS); Cancer-associated retinopathy (CAR); paraneoplastic opsoclonus-myoclonus ataxia (POMA); paraneoplastic cerebellar degeneration (PCD); paraneoplastic neurological syndromes (PNS); Paraneoplastic neurologic disorder (PND); Paraneoplastic stiff man syndrome (SMS); Not determined (ND).

The nomenclature of SCLC-associated antibodies and antigens has evolved over time, largely because the specific antigens were originally unknown, and the antibodies were classified based on reactivity with whole cells or whole cell lysates. For example, ANNA (anti-neuronal nuclear antigen) reactivity was later reclassified as anti-Hu to reflect the fact that these antibodies target the Hu proteins [23-26]. Currently, there are at least 20 known antigens in SCLC-associated PNS. Interestingly, patients are often positive for more than one SCLC-associated autoantibody [27-32], and may have several paraneoplastic syndromes [33,34]. The possible mechanisms causing immunoreactivity as well as the possible pathogenesis of SCLC-related autoimmunity will be discussed later in this review.

The SCLC-associated autoantigens/autoantibodies identified to date are summarized in Table 1. Three different types of SCLC-associated PNS and their target autoantigens are discussed in more detail below as examples: 1) paraneoplastic encephalomyelitis/sensory neuronopathy (PEM/SN), in which Hu proteins are a target, 2) Lambert-Eaton myasthenic syndrome (LEMS), in which voltage-gated calcium channels (VGCC) and SOX proteins are targets, and 3) cancer-associated retinopathy (CAR), in which recoverin is a target.

### Paraneoplastic encephalomyelitis sensory neuronopathy (PEM/SN) and the Hu antigens

Anti-Hu autoantibodies were first detected in SCLC patients with subacute sensory neuropathy [23-25,35]. Serum from a SCLC patient with PEM/SN was used to screen a lambda cerebellar expression library, which led to the identification of an antigen that was named HuD [36]. HuD belongs to a family of four related RNA-binding proteins. These proteins are homologous to the *Drosophila melanogaster *protein Elav (embryonic lethal, abnormal visual) which plays a role in eye and central nervous system development of the fly [36]. Expression of three of the Hu proteins--HuB/Hel-N1/ELAVL2, HuC/ELAVL3, and HuD/ELAVL4--is restricted to the nervous system and gonads, while expression of the fourth, HuR/ELAVL1, is ubiquitous [37]. In vertebrates, neuronal Hu proteins play a role in neuron-specific RNA processing and neural development [36,38-40].

About 85% of PEM/SN patients have underlying SCLC, and these patients harbor high titers of autoantibodies [26,41,42] that react strongly against all three neuronal Hu proteins and weakly against HuR [24,26,43,44] (Table 1). Hu proteins contain three RNA recognition motifs (RRMs). A short presumably unstructured N-terminal region precedes RRM1, followed by RRMs 2 and 3, which are separated by a hinge region. Using Hu deletion constructs, the pattern of anti-Hu reactivity has been examined in anti-Hu positive PEM/SN patients with SCLC [45,46], in mice immunized with full-length HuD [46], in SCLC patients with and without PEM/SN [47], and in a SCLC mouse model system [48]. Consistent reactivity against the RRM1-containing N-terminal region of the protein was common among all of the studies, suggesting RRM1 may be the key target of the autoimmune response.

**Figure 1:**
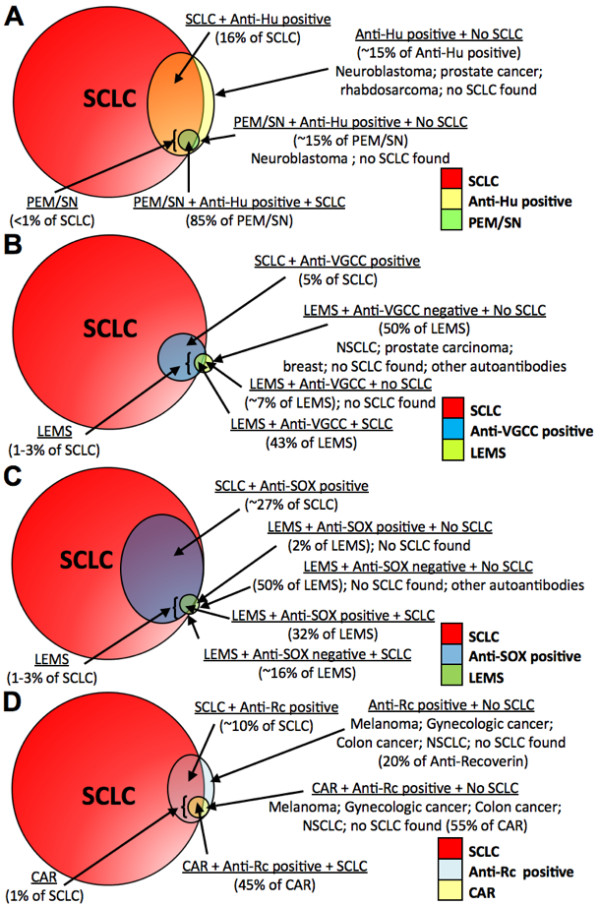
**Association between SCLC, paraneoplastic syndromes, and (A) anti-Hu, (B) anti-voltage-gated calcium channels (VGCC), (C) anti-SOX1, and (D) anti-recoverin (Rc) autoantibodies**. Arrows provide further description of distinct groups within subsections of each circle or oval (see Table 1 for references).

All SCLC tumors express neuronal Hu antigens; however, PEM/SN presents itself in less than 1% of all SCLC patients [36,41]. It is unknown why a small fraction of patients develop the autoimmune disease while the vast majority does not. Interestingly, 16-25% of SCLC patients *without *paraneoplastic neurological autoimmune syndromes have detectable titers of anti-Hu antibodies in their serum, albeit at much lower levels than PEM/SN patients (Table 1, Figure 1A) [26,49-52]. While the frequency of anti-Hu antibodies in SCLC is too low to be of value for SCLC diagnosis or early detection, one could envision that a panel of SCLC-associated antigens will increase sensitivity.

### Lambert-Eaton myasthenic syndrome and the voltage-gated calcium channel and SOX1 antigens

Lambert-Eaton myasthenic syndrome (LEMS) is an autoimmune disorder of the neuromuscular junction and is characterized by muscle weakness, absence of reflexes, and autonomic dysfunction [53]. An underlying SCLC tumor is found in up to 50% of LEMS patients [54], and discovery of SCLC can occur more than five years after diagnosis of the autoimmune disease [55]. It has been difficult to determine whether the tumor precedes the neurologic disorder or vice versa, but the diagnosis of LEMS initiates a tumor search based on the high degree of correlation between LEMS and SCLC [56,57], and the detection of various autoantibodies has aided in cancer diagnosis. Multiple antigens are associated with LEMS, and some LEMS patients with SCLC will exhibit one or multiple autoantibodies (Table 1), including anti-voltage-gated calcium channel (VGCC) and anti-SOX1.

SCLC tumors ectopically express functional neuronal presynaptic P/Q-type voltage-gated calcium channels (VGCCs) [58](Figure 1B). These are among five types of high voltage-activated neuronal calcium channels: L, N, P, Q, and R [59-61]. The α-1, α-2, β, and δ subunits make up this multimeric transmembrane protein, and it has been reported that the α-1 subunit may contain the antigenic epitope to which anti-VGCC antibodies bind [62-64]. A key aspect of the antibodies against the P/Q-type VGCC is that they are able to bind to extracellular targets [65] and have been shown to disturb calcium-dependent transmission across the neuromuscular junction [66], indicating that they may play a direct role in the pathogenesis of LEMS. Additional evidence supporting this hypothesis include: 1) LEMS IgG reduced the number of VGCCs on cultured SCLC cell lines [67-69], 2) treatment of SCLC tumor (radiation therapy, chemotherapy, or resection) was followed by clinical remission of LEMS [65], and 3) mice injected with LEMS IgG displayed physiological and morphological abnormalities similar to the human disease [70-72]. Similar to the Hu proteins, it is believed that the misexpression of neuronal VGCCs in SCLC initiates an immune response. In the case of LEMS, the antibodies are thought to cross-react with VGCCs in cholinergic nerve terminals at the neuromuscular junction and in the cerebellum [66,73,74]. Importantly, patients with the idiopathic form of LEMS (NT-LEMS) also harbor high titers of anti-VGCC autoantibodies [75-77], making it difficult to distinguish between paraneoplastic and nonparaneoplastic LEMS. This calls into question the potential utility of this particular type of antibody for diagnosis or early detection of SCLC, since it would lower the specificity of any resulting test.

Recently, anti-glial nuclear antigen (anti-AGNA) antibodies have been described as among the many autoantibodies associated with LEMS [78]. Though their role in the pathogenesis of the paraneoplastic disease remains unknown, the antibodies present themselves in a large percentage of SCLC patients, more so than most well characterized autoantigens (see Table 1). The antibodies bind to the nuclei of Bergmann cells, a specialized glial cell type exclusively found in the cerebellar cortex [79]. Screening a fetal brain library with AGNA-positive sera from SCLC patients led to the identification of SOX1 (Sry-like high mobility group box) as an antigen of anti-AGNA positive patients with SCLC-associated LEMS (Table 1) [30,80]. The SOX family of DNA-binding transcription factors is important in the development of the nervous system [81] and contains a highly conserved HMG-box DNA-binding domain. Of all LEMS patients with confirmed SCLC, approximately 64% have detectable titers of anti-SOX1 antibodies (Figure 1C). Idiopathic LEMS cases (without SCLC) rarely show positive reactivity to SOX1 and no anti-SOX antibodies have been detected in healthy controls, indicating that the SOX1 autoantibodies may be highly specific for SCLC [30,31]. In the SCLC patient population without LEMS, up to 36% harbor antibodies to one or more SOX protein family members (Table 1). Thus, inclusion of SOX antigens in a SCLC antigen panel could increase sensitivity and specificity of a future antibody test aimed at SCLC diagnosis or early detection.

### Cancer-associated retinopathy and the recoverin antigen

Cancer-associated retinopathy (CAR) is characterized by the degeneration of photoreceptors in the presence of cancer, leading to blindness. A 26 kDa retinal protein was identified as a key component in the immunologic events that accompany CAR [82]. Serum antibodies from CAR patients with concurrent SCLC were used to isolate the corresponding gene by screening against a cDNA library made from human retina [83]. Sequence analysis revealed high homology with the bovine photoreceptor protein recoverin [84]. Recoverin, a calcium-binding protein in the retina, is involved in guanylate cyclase activation and function; guanylate cyclase is an essential component of the phototransduction cascade initiated by rhodospsin [85]. It has been shown that antibodies directed against recoverin can neutralize its activation of guanylate cyclase [85], which could, in turn, interfere with photoreceptor function and lead to the loss of photoreceptors, which is characteristic of CAR [86]. Thus, these SCLC-associated autoantibodies might play a role in the autoimmune pathology.

Just as with anti-Hu reactivity, anti-recoverin (anti-Rc) antibodies can also be found in SCLC patients without paraneoplastic disease (Figure 1D). In an analysis of tumor tissues and sera from 143 patients with lung cancer, 68% of SCLC tumors expressed recoverin while 15% of sera from these patients were positive for anti-Rc antibodies. None of the 143 patients had CAR [87]. The presence of anti-Rc antibodies in a substantial fraction of SCLC patients suggests that recoverin might show promise for SCLC detection as part of an antigen panel.

## Induction of immuno-responsiveness in SCLC

The mechanisms that trigger and maintain an autoimmune response in cancer patients are poorly understood. An autoimmune response is a specific immune response to a self-antigen, and autoimmune diseases occur because the immune system is incapable of discriminating between self-antigens that are normally expressed and those that are abnormally or ectopically expressed. Some SCLC-associated autoantigens, such as the Hu and SOX1 proteins, are intracellular antigens. The following sections will discuss various hypotheses concerning the induction mechanism of immuno-responsiveness against self-antigens, including abnormal expression of proteins, tolerance disruption, cross-reaction with membrane antigens, and protein modifications that generate "neo-antigens".

### Abnormal expression of self-antigens and the immune privilege of neurons

SCLC-associated autoimmunity may arise as a consequence of abnormal self-antigen expression by tumor cells. Ectopic expression of a self-antigen alone is not sufficient to initiate an immune response; the immune system must somehow be activated. It is understandable that a mutation or modification of a self-antigen could render it foreign; however, if this is not the case, another explanation must exist. Many tumor-associated antigens are not unique to tumor cells. Most SCLC-associated antigens are normally expressed in the nervous system and gonads, and the tumor antigen and natural antigen appear to be identical. In these cases, the antigen may present in a way that the immune response is unaccustomed to, either at an increased level of expression or altered localization, which could be detected by the immune system, initiating an immune response.

An important question to address is why only a fraction of patients harbor an immune response when many of the antigenic proteins are aberrantly expressed in almost all SCLC tumors? For example, 16-25% of SCLC patients have an anti-Hu response without neurological disease [26,31,49,52,88] even though all SCLC tumors express the antigen [89]. This suggests that those cells expressing the antigen may be immune privileged, allowing evasion of an immune response. A recent study found that HuD-specific CD8+ T cells were normally present in C57BL/6 mice, but these T cells did not expand upon immunization with an adenovirus expressing HuD. The T cells were stimulated only when the splenocytes from these immunized mice were stimulated *in vitro*. These cells were able to recognize the antigen *in vivo*, but were prevented from becoming effector cytotoxic T cells, suggesting that the mice were strongly tolerant to the self-antigen HuD protein. Furthermore, after HuD immunizations or HuD CD8+ T cells through adoptive transfer, these mice did not develop evidence of neurologic dysfunction or abnormality. Further evidence for tolerance to HuD comes from experiments with HuD null mice, where HuD is not a self-antigen. These mice generated functional HuD-specific T cells, and these cells were directly activated without the need for *in vitro *stimulation with a peptide [90]. Taken together, these results demonstrated a robust tolerance to HuD *in vivo*.

Most SCLC-associated autoantigens are normally expressed in a neuron-specific manner. Neurons do not normally express major-histocompatibility complex (MHC) class I molecules [91], thus they may be immune privileged cells that can evade immune surveillance. It was observed that some SCLC tumors from patients with PEM/SN did express MHC class I molecules, whereas amongst 20 tumors from patients with detectable anti-Hu autoantibodies and no PEM/SN, only one showed modest expression of MHC class I molecules [41]; tumors from most seronegative patients did not express MHC proteins. Together, these studies indicate that tolerance to neuronal antigens may be present, and that its disruption might contribute to SCLC-associated autoimmunity.

### Modifications of self-antigens to generate "neo-antigens"

An alternative hypothesis for the autoimmune response in SCLC is that the antigenic protein might be mutated or modified in a way that renders it foreign to the immune system. It is possible that the tumors express similar, but not identical forms of the antigen. For example, mutations or alternative splicing may generate different forms of the protein. In a recent study, the HuD sequence of four SCLC tumors and five SCLC cell lines was determined [92]. A missense mutation was identified in two tumors, but it was unclear whether the mutations were single nucleotide polymorphisms (SNPs) or somatic mutations. None of the cell lines showed HuD mutations, although several had SNPs. A previous study of 18 SCLC cell lines, including one from the tumor of a patient with PEM/SN, also failed to identify mutations or rearrangements in HuD [93]; the same was true in an analysis of paraneoplastic SCLC tissue [94]. However, these studies did not examine the related genes HuB and HuC, which are often misexpressed in SCLC and to which anti-Hu autoantibodies are cross-reactive. No mutations were found in the cerebellar degeneration-related (cdr2) gene, which encodes a neuron-specific protein in breast and ovarian tumors associated with PCD [95]. Overall, there is little evidence for the involvement of mutations in SCLC-associated autoimmunity, however further studies are needed to eliminate this as a potential factor.

Another possible explanation for immunogenicity of SCLC-associated autoantigens is that proteins triggering the autoimmune response might carry abnormal post-translational modifications. Over 140 unique amino acids and amino acid derivatives are reported to exist in proteins as a result of post-translational modification [96]. Such modifications include glycosylation, phosphorylation, methylation, acetylation, deamidation, and isomerization (reviewed in [97,98]). These modifications can be enzyme-mediated or spontaneous. Abnormal post-translational modifications can lead to the generation of "neo-self" antigens which the immune system recognizes as foreign and to which it mounts an immune attack. For example, experimental autoimmune encephalomyelitis (EAE) is only induced in a murine model for human multiple sclerosis when mice are immunized with an acetylated N-terminal peptide of myelin basic protein (MBP-Ac1-11); no T cells are stimulated and no EAE is elicited upon immunization with the non-acetylated form of the peptide [99]. A number of autoimmune diseases, including multiple sclerosis, experimental allergic encephalomyelitis, systemic lupus erythematosus, and rheumatoid arthritis, are associated with post-translational modifications (reviewed in [96,98,100]). Recently, changes in IgG Fc N-glycosylation were observed in LEMS and myasthenia gravis (MG) patients [101]. Antibodies elicited as a result of modified self proteins are often able to bind both the modified and unmodified form of the protein, possibly by epitope spreading; however, the T cell response is usually specific for the modified form, retaining tolerance for the normal protein [97].

To our knowledge, the association between post-translational modifications of SCLC-associated autoantigens and SCLC-related autoimmunity has not been examined. It is possible that post-translational modification of neuronal proteins in SCLC tumors initiates an immune response which may cross-react with native proteins in the healthy nervous system and, in extreme cases, leads to paraneoplastic disease. Therefore, the role of post-translational modification as a trigger of SCLC-associated autoimmunity should be thoroughly investigated.

## Pathogenicity of SCLC-associated autoimmune responses

Understanding the pathogenesis of SCLC-associated PNS is important to elucidate the mechanism by which the immune response arises as well as to clarify the potential limitations of harnessing the immune system to fight cancer. If mutated or aberrantly modified proteins originating from the cancer trigger the SCLC-associated immune responses then one might expect the response to be tumor-specific. How would reactivity spread to the native protein and lead to destruction of healthy tissue? One mechanism that has been proposed is epitope spreading [97,102]. Epitope spreading is the gradual expansion of the spectrum of specificities recognized in B and/or T cell-mediated immune responses. Evidence for epitope spreading has been demonstrated by immunization experiments with a single peptide derived from an autoantigenic protein, giving rise to a T cell response or autoantibody production directed against epitopes that did not overlap with the immunizing peptide [102]. Analysis of B and T cell reactivity in autoimmune diseases does, however, suggest that the T cell response is frequently specific for the modified antigen, while the B cell response is more cross-reactive [97]. This may be related to the mechanism by which antigens are displayed by MHC molecules and recognized by the T cell receptors [97]. In SCLC-associated autoimmunity, multiple epitopes are often recognized within a single antigen [91,103], also supporting a role of epitope spreading in the pathogenicity of SCLC-associated paraneoplastic syndromes.

The tissue naturally expressing SCLC-associated autoantigens will affect the pathogenesis of PNS. In the case of SCLC-related autoimmunity, most of the autoantigens are neuronal; thus, certain parts of the nervous system would be targeted and affected. In addition, the cellular localization of the antigen in its natural context will also play a role. Synaptic proteins, for example, are more likely to be expressed on the cell surface [104], and a pathogenic role of autoantibodies against these antigens is therefore more likely than of antineuronal antibodies directed against intracellular proteins. Cell surface antigens can be directly recognized by antibodies and CD8+ T cells, which would result in a directed immune attack and subsequent apoptotic cell death of the offending cell. Apoptotic cells can present altered cleavage products and post-translationally modified self-proteins on surface blebs, further promoting autoimmunity [97].

Antibody binding may also affect the function of the target protein. In SCLC-associated LEMS, voltage-gated calcium channels (VGCCs) are found both in the tumor and the presynaptic cholinergic-synapse and cerebellar Purkinje cells of the nervous system [66]. VGCCs are transmembrane proteins, and antibodies reacting with them in the tumor and the nervous system bind and disrupt the normal structure of the extracellular protein, possibly contributing to the development of LEMS. Anti-Nova antibodies (Table 1) have been shown to play an inhibitory role on Nova-1 binding to its RNA target [105], suggesting that the antibodies may disturb the function of the protein. Whether this biological effect contributes to the formation of paraneoplastic opsoclonus-myoclonus ataxia (POMA) is unknown.

It has been proposed that antibodies or cytotoxic CD8+ T cells can cross-react with antigens in the nervous system by penetrating the blood-brain barrier--a physical barrier separating the central nervous system from systemic circulation and restricting the passage of solutes into cerebrospinal fluid. These antibodies and T cells can then bind to the antigen expressed on the neurons and impair neuronal activity, triggering apoptosis [22]. For example, it has been hypothesized that anti-recoverin antibodies present in peripheral blood can penetrate the blood-retina barrier and become internalized into photoreceptor cells expressing recoverin, which could block recoverin function and lead to photoreceptor cell death [106-108]. Some onconeural antigens, such as Hu proteins, however, are normally expressed in the peripheral nervous system neurons where the blood-brain barrier does not exist [109]. Thus, an alternate model is likely in those cases.

While there is ample evidence for a role of antibodies in the pathogenesis of PNS, there are also examples of high titer antibodies with no overt neurological effect. For example, when mice were immunized with a HuD DNA or HuD protein vaccine, no neurological disease was observed even though an antitumor response that inhibited the growth of an implanted neuroblastoma was observed. Furthermore, passive transfer of IgG from anti-Hu patients to animals did not induce an autoimmune response [110,111]. This suggests that a correlation exists between antitumor immunity and the presence of the antibodies, but that the antibodies are not causing the autoimmune disease. Thus, the pathogenicity of SCLC-related autoantibodies may be dependent on the antigen and its effect on its target cell. Cellular immunity has been shown to play a role during the course of SCLC-associated PNS. Several groups have reported a T cell response in anti-Hu positive patients, and a HuD-specific cytotoxic T cell response has been implicated in the development of PEM/SN [112-115]. A subpopulation of nontoxic T cells was also identified in PEM/SN patients, indicating that both classical cytotoxic T cells and noncytotoxic T cells may play roles in pathogenesis [114]. In seeming contrast to these observations, one group showed no evidence of HuD-specific T cells in the cerebrospinal fluid of SCLC-associated PEM/SN patients [116,117]. However, the authors noted that the assay used may not have been sufficiently sensitive. Thus, the role of antigen-specific T cells in the pathogenesis of SCLC-associated autoimmunity is still under debate. It has been shown that major-histocompatibility complex (MHC) class I and MHC class II antigen-presenting molecules in neurons can be recognized by T cells which can kill neurons [118]. Intracellular proteins can be presented to T cells on the surface of MHC class I molecules. However, neurons do not normally express MHC class I molecules [91], indicating that these cells may be immune privileged, allowing evasion of T cell recognition.

Recently, attention has turned to the role of CD4+ CD25+ regulatory T cells (T_reg_), which are suppressor cells that maintain immune tolerance. T_reg _populations generally increase in and around cancer tissues, which potentially causes the down regulation of both effector T cell function and antitumor immunity, thereby contributing to cancer growth [119,120]. However, LEMS and PEM/SN patients exhibited a down regulation of these T cells in comparison to SCLC patients without LEMS or PEM/SN [121], suggesting T_reg _dysfunction may play a role in the PNS development.

In summary, many questions remain about the pathogenicity of the SCLC-associated immune response, and the possibility that distinct mechanisms play a role in different paraneoplastic syndromes further increases the complexity of this disease state. Various hypotheses about the ectopic expression of autoantigens, the molecular state of antigens themselves, and the role of the immune system and the pathogenicity of antibodies and T cells are currently under study. Animal models are a promising tool for testing these various hypotheses and helping to elucidate the pathogenesis of autoimmunity as well as the autoimmune trigger.

## Animal models as tools for understanding the etiology of SCLC-associated autoimmunity

As outlined above, the underlying causes of SCLC-related autoimmunity and how it leads to pathology remain poorly understood despite many years of investigation. The rapid progression of SCLC coupled with the rarity of the related autoimmune diseases make studies in human patients challenging. Animal models can be extremely helpful for the investigation of disease development and progression; they allow many analyses that are difficult or impossible to carry out in human patients.

Generating animal models that accurately represent human SCLC-associated paraneoplastic neurological syndromes has been very challenging. For example, the passive transfer or intraventricular injection of anti-Yo antibodies from patients with PCD (Table 1) into mice and rats did not induce disease in the animals [122,123]. When HuD was used as either a protein or DNA to immunize mice, a strong anti-Hu response and high titers of T cell inflammatory infiltrates were observed in all immunized animals [110,111]. Although some insight into the anti-Hu immune response was gained from these studies, these animals did not develop neurological symptoms or neuropathological abnormalities. The transfer of autoreactive T cells against the PNMA1 antigen in rats resulted in no clinical signs of neurologic disease despite an inflammatory response in the brains of the animals [124]. More recently, two mouse models with immunologically induced retinopathy associated with elevated recoverin antibodies were developed. In one case, mice were immunized with recombinant recoverin three times. In the other, mice were injected with hybridoma cells that produce a monoclonal antibody targeting recoverin. In both models, retinopathy was observed, suggesting that this kind of approach can be used to mimic this particular type of autoimmunity [125]. In another recent study, stiff person syndrome-like symptoms were induced in rats through repetitive intrathecal application of anti-amphiphysin IgG antibodies, including stiffness of trunk and limb muscles, muscle spasms, and gait abnormalities [126]. One weakness of all the aforementioned models is that immunogenicity was induced by exogenous introduction of antigens or antibodies and not by the "natural" presence of SCLC. The development of a SCLC animal model and the study of its associated immune response would provide a better model system for SCLC-associated PNS.

Creating a SCLC animal model has not been easy. Expression of proneural transcription factor human achaete-scute homolog-1 (hASH-1), which is highly expressed in SCLC, under the control of the bronchial epithelial-expressed Clara cell CC10 promoter in mice was not successful. Even though these mice exhibited rapid hyperplasia after being crossed with a transgene CC10-SV40 large T antigen, they developed adenocarcinomas that did not resemble human SCLC [127]. Other attempts have been made using xenograft models with SCLC cell lines or primary SCLC tumors [128-130]. However, these strategies require immune-compromised animals, thereby limiting their utility for studies of autoimmunity. Success was finally achieved through the conditional knockout of the *Rb *and *Trp53 *genes in the lungs of mice [131,132]. The inactivation of both genes is commonly found during lung cancer pathogenesis and has been identified in up to 90% of human SCLCs [4,133]. Using a Cre-loxP system, "floxed" *Rb1 *and *Trp53 *can be homozygously deleted in the lung epithelium of transgenic mice through intratracheal instillation of Adeno-Cre virus. All treated mice develop multiple tumors with histopathology and immunophenotype similar to human SCLC beginning around 200 days post infection [131,132]. The prolonged lag time allows for the monitoring of potential immune responses against various SCLC-related autoantigens prior to the clinical detection of the disease. Recently, it was observed that the loss of *p130*, a cell cycle inhibitor related to *Rb *[134] that normally suppresses SCLC development [135], accelerates the development of SCLC in *Rb*/*Trp53*-mutant mice. *Rb*/*Trp53*/*p130 *mutant mice may thus provide an alternative mouse model of SCLC with a shortened lag time [136].

Our laboratory examined the anti-Hu response in the inducible *Rb/Trp53 *knock out mouse model system [48]. Just like their human counterparts, tumors derived from the SCLC mouse model expressed Hu proteins. Interestingly, elevated anti-Hu antibodies were detected in 14% of SCLC-prone mice, similar to the frequency of above background anti-Hu response in human SCLC patients. Furthermore, the pattern of reactivity against the Hu protein family and Hu deletion constructs was similar to that observed in human patients [45-47], supporting the notion that the N-terminal part of the protein containing RRM1 may contain the epitopes that bind to MHC and may be the key target of the autoimmune response. Thus, this mouse model system closely mimics previously observed aspects of the anti-Hu response in human SCLC patients.

**Figure 2:**
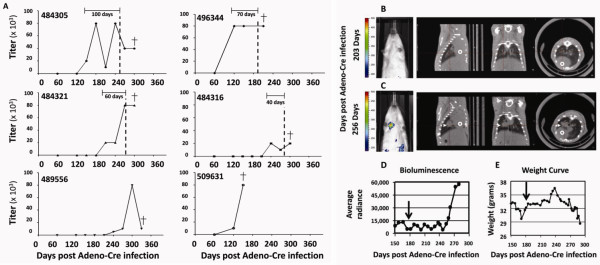
**Anti-Hu reactivity relative to SCLC detection and image-based screening of SCLC-prone mice**. **(A) **Anti-Hu reactivity was detected 40-100 days prior to clinical detection of SCLC. Graphs from the six highly positive mice are shown. The dotted line represents the date of first clinical detection of tumor by CT scan and/or luminescence detection. The titer was determined by the highest fold dilution of plasma or serum in which there was a positive Western blot signal against recombinant HuD protein. The time (in days) between the measurement of a titer above background (titer > 5000) and clinical detection is indicated by a horizontal bar. SCLC tumor was not detected in mouse 489556, though the mouse became very sick. Mouse 509631 was sacrificed at the time of high titer detection, and the lungs of this animal showed neuroendocrine lesions *in situ*, but cancer was not detected. Crosses indicate dates of sacrifice. Titer values are in thousands. (**B-E**) Mice carrying homozygously floxed p53 and Rb alleles, as well as a luciferase reporter under the control of a β-actin promoter, were infected with adenovirus carrying the Cre recombinase gene via intratracheal instillation. Mice were monitored for tumor formation using bioluminescence detection (**B**, **C**, left panels, and **D**), X-ray based CT-scan (**B **and **C**, right panels, open circles, heart; arrowheads, tumor mass), and clinical symptoms like weight loss (**E**), altered coat, or kyphosis. **B-E **show the follow up of mouse 484305 which showed anti-Hu reactivity with a titer of 80,000 at 180 days (arrows) after Adeno-Cre infection. At that time, tumor was undetectable by imaging techniques (images taken 203 and 256 days after Adeno-Cre infection) (**B**). Tumor became detectable 256 days after Adeno-Cre infection (**C**). Acute weight loss was observed 286 days after Adeno-Cre infection (**E**). (Reprinted from [48], with permission from Elsevier, License Number 2460341062344).

The inclusion of a recombination-induced luciferase gene in the SCLC model system enables cancer development to be monitored by bioluminescence as well as by small animal computed tomography (CT). This allows for the timing of the anti-Hu response relative to the clinical detection of the tumor to be measured. Initial studies with a limited number of animals indicated that anti-Hu antibodies could arise up to 100 days before the cancer was clinically detectable (Figure 2). In this initial published study, no neurological tests were performed to determine whether the mice showed signs of paraneoplastic disorder. While an antibody response is detected in 16-25% of human SCLC patients, manifestation of PEM/SN is rare. If the mouse model parallels the human situation, PEM/SN will be much less frequent than an above background anti-Hu antibody response. If PEM/SN ever develops in the context of this model, this will likely require the analysis of a large number of mice. However, even if PNS were rare in the SCLC mouse, the development of an anti-Hu antibody response in a substantial fraction of the mice suggests the SCLC mouse model should lend itself well to the study of immune responses against other SCLC-associated paraneoplastic antigens and to the elucidation of the mechanism of SCLC-associated autoimmunity.

The SCLC mouse model may also shed light on whether the induced autoantibodies antagonize cancer progression. This is still a topic of debate in human patients [28,31,41,42,50,52,88,137-144]. In humans, some studies have noted a correlation between the presence of the autoantibodies and indolent tumor growth [42,88,138]. Interestingly, in our initial small study with SCLC-prone mice, we did observe one mouse that was highly positive for anti-Hu autoantibodies yet lacked an overt tumor [48]. While we did not detect a survival benefit of an anti-Hu response in the initial mouse study, we estimate that about 180 mice would be needed to clearly show the presence or absence of an effect of the anti-Hu response on survival [48].

In conclusion, the SCLC mouse model offers a highly promising new window onto the development and consequences of SCLC-related autoimmunity. To date, only anti-Hu reactivity has been examined in this model system, and it will be of great interest to test these mice for other types of antibodies, such as those listed in Table 1. Most importantly, the SCLC-prone mouse model will lend itself to mechanistic studies of the immune response and its timing relative to cancer onset. It will also be a great tool to examine potential clinical applications of autoantibodies and molecules targeting the autoantigens, such as imaging and therapeutic agents.

## Potential clinical applications of SCLC-related autoantibodies and their antigens

### Early detection and screening strategies for SCLC

Effective early detection of any cancer (i.e. detection that allows complete removal of lesions before they can metastasize) has the potential to greatly reduce cancer-related mortality. Ideally, this would be achieved by non-invasive molecular imaging combined with biomarker-based tools [145]. An effective screening method must have high specificity and sensitivity, reduce overall mortality, be acceptable to patients, and be cost effective in order to be widely adopted [146].

To date, imaging has shown limited promise for the early detection of SCLC. [^18^F] fluorodeoxyglucose positron emission tomography (FDG-PET) scanning, a method that has been used to detect SCLC tumors in paraneoplastic patients positive for anti-Hu antibodies, has not been shown to decrease mortality [147]. This suggests that FDG-PET is unable to detect SCLC lesions in time for curative resection. Low Dose Spiral Computed Tomography (LDSCT) is under evaluation as an early lung cancer detection modality. Studies from the Early Lung Cancer Action Project (ELCAP) suggest that annual spiral CT screening can detect lung cancer at a curable stage and increase lung cancer survival [148-151]. However, any early detection method causes lead time bias; the favorable survival noted in the ELCAP study is therefore not a valid measure of the efficacy of screening [152]. In addition, LDSCT was shown to incorrectly classify many non-calcified nodules as cancers [153,154], potentially subjecting patients to unnecessary follow-up procedures (scans, biopsies, or resections), which are costly, invasive, and can result in patient morbidity and mortality [149,155,156]. Recently, it has been hinted that the National Lung Cancer Screening Trial, a randomized control trial of high risk long term smokers subjected to either LDSCT or X-ray based screening [157], might show a reduction in mortality in the LDSCT-screened group. The published results of the study are therefore anxiously awaited so that the potential benefits and drawbacks of screening by LDSCT can be weighed and the guidelines for the use of this tool can be established.	 Whether this type of screening could benefit SCLC patients is unclear. Early detection of SCLC through imaging is especially challenging because SCLC can metastasize when the primary lesion is still very small. Small tumors are more challenging to detect because they generate less signal. In addition, if SCLCs are small at metastasis, imaging-based screening may allow too little time between detection and metastasis for successful intervention. Thus, it is unlikely that imaging alone will be of use for early detection of SCLC. Some kind of molecular marker will likely be required. However, if a molecular assay depended on nucleic acids or protein molecules derived from the tumor, the same problem of tumor size would apply, since these molecules may be secreted into the blood in undetectable amounts when the tumor is small. This caveat may not pertain to an antibody response; it is unknown what size a SCLC tumor must be to trigger immune reactivity.

Autoantibodies against tumor-associated antigens are more stable and specific than other serum-derived proteins [158], making them good candidate biomarkers for cancer detection. Because the natural history of SCLC is unknown, it is possible that small tumors or precancerous lesions may be present in patients for a long time before the tumors develop metastatic abilities. Many patients have smoked for decades before they develop SCLC. Depending on the proteins they express, preneoplastic lesions might trigger an immune response. In SCLC patients with paraneoplastic disease, the immune response is frequently noted before the tumor is detected [75]. It is, however, unclear if the timing of events is similar in SCLC patients *without *PNS. In our studies of the immune response in SCLC-prone mice, we observed one mouse with high titer antibodies and *in situ *neuroendocrine lesions, but no invasive cancer [48]. This, and other observations from the SCLC mouse model, support the notion that an immune response can precede clinical detection (Figure 2). However, the window of detection in the mouse model is small (several months at best). It remains to be seen how the timing of events in this genetically engineered mouse model system translates to the human situation in which (epi)genetic alterations, potentially occurring over the course of many years, result in the development of SCLC. Despite this drawback, at this time, the SCLC mouse model system offers the best tool to gain insight into the temporal relationship between SCLC development and the immune response. Studies of a larger number of mice, including analysis of their lungs as elevated titers of antibodies develop, will hopefully shed light on the type of lesions that are present when the autoimmune response is triggered.

Identifying biomarkers highly *specific *for a malignant condition is one great challenge in developing a serological detection tool. In the case of autoantibodies, multiple environmental factors, pathogen invasion, and autoimmune disease can result in the production of a high-level of IgG and IgM autoantibodies that recognize various antigens and thus reduce the specificity of the antibody [159-161]. The prevalence of smoking in healthy individuals may also affect their immune response (reviewed in [162]). In addition, the detection of background reactivity may be influenced by technology; the more sensitive the technique, the greater the probability that background reactivity will be detected. A recent study analyzed a panel of tumor-associated autoantibodies in 205 healthy individuals and found a subgroup of individuals that showed elevated levels of immunoreactivity against most of the antigens tested [163]. Unfortunately, no SCLC-associated antigens were examined in this study. In another analysis, the presence of anti-Hu reactivity was examined in plasma from 120 subjects, including 79 healthy controls (smokers and non-smokers) and 41 SCLC cases [52]. The latter was a population-based study matched on age, race, sex, and smoking status in which anti-Hu reactivity was examined in relation to lung cancer risk. Although anti-Hu reactivity was found to be significantly higher in cases than controls, low level anti-Hu reactivity was detected in healthy non-smokers (~39%) as well as smokers (~44%). Dalmau and colleagues also observed anti-Hu reactivity in non-cancer individuals; they considered reactivity in normal subjects as background and used it as cut-off to score anti-Hu reactivity in SCLC patients with and without PEM/SN [26]. In other studies, reactivity of anti-VGCC autoantibodies in normal controls was used as a cut-off for positive reactivity [66,164]. The presence of background reactivity in non-cancer patients will likely limit the specificity of any anti-Hu antibody-based SCLC test, and will require the stringent determination of cut-off values for reactivity considered positive.

Background reactivity does not appear to occur for all antigens. In a study examining anti-recoverin response, no healthy individuals showed positive reactivity [87]. However, SCLC is not the only cancer associated with anti-recoverin antibodies, which can also be found in several other cancers (Figure 1). No anti-SOX1 antibodies were found in healthy individuals in another study testing the sera of 27 healthy blood donors [165]. These examples illustrate that careful choices must be made when considering which SCLC antigens might be useful for early detection tests.

The second big challenge in developing a serological detection tool is to attain sufficient *sensitivity*. It is clear that a detection test utilizing any single SCLC antigen will show insufficient sensitivity because any single antibody is present in only a fraction of SCLC patients (Table 1). However, examination of autoantibody reactivity in SCLC patients shows that different patients can exhibit distinct immune responses and in some occasions, an immune response against multiple antigens [29,31,166,167]. Thus, a *panel *of SCLC-related autoantigens may provide the necessary increased sensitivity, as was seen, for example, by Titulaer and colleagues when examining four SOX family members and the three neuronal Hu antigens in SCLC patients with and without LEMS [31]. The authors from this study find 67% sensitivity and 95% specificity in discriminating between SCLC patients with LEMS and LEMS patients without tumors when testing against a panel of the SOX family members (Sox 1, 2, 3, and/or 21) [31]. This latter study underlines the need to continue searching for new SCLC-associated autoantigens.

Will a combination of antigens ever attain the required sensitivity? To try to answer this question, we can examine two extreme hypothetical situations. In one, all patients show non-overlapping immune responses against different autoantigens. In this case, a panel may greatly increase sensitivity; the sum of the percentages of all SCLC patients immuno-positive for currently known antigens would allow detection of SCLC in 100% of patients (see Table 1). In the opposite scenario, immunoreactivity is only ever present in the same small subset of SCLC patients that could exhibit a wide variety of antibodies. In this case, the sensitivity one could achieve using a panel of antigens might be as small as 25%. The latter scenario is unlikely. For example, no correlation between the presence of anti-Hu and anti-VGCC autoantibodies was found in 200 newly diagnosed lung cancer patients. Although four patients were positive for both autoantibodies, individually, up to 25.5% and 5% were immuno-reactive against Hu proteins and VGCC, respectively [50]. Thus, the combination of carefully selected antigens may ultimately provide a marker panel that could be of use for early SCLC detection. Indeed, recent studies show that sensitivity and specificity is greatly enhanced when a combination of antibodies is used to detect lung cancer [31,168,169]. Aside from careful choices of markers, defining an at-risk group that could be screened for early disease would be crucial to increase the feasibility of any test. In the case of SCLC, longtime heavy smokers would be prime candidates for screening. When examining anti-Hu reactivity in smoking and non-smoking healthy controls [52], one of the smoking controls with detectable anti-Hu reactivity and healthy at the time of the study died of SCLC several years after the conclusion of the study. Based on the expected frequency of lung cancer in this group and the relatively minor contribution of SCLC, the SCLC case is remarkable. However, such incidental findings are impossible to interpret. In addition, three highly anti-Hu positive cases did not develop SCLC within the time frame of the study. Interestingly, one of the anti-Hu positive cases died of prostate cancer, which can occasionally exhibit anti-Hu antibodies (Figure 1A) and can show a neuroendocrine "small cell" type [89].

It is clear that questions of sensitivity and specificity of a combined panel of SCLC-associated autoantigens for detection of SCLC will require further study. A retrospective study using plasma/serum collected from a lung cancer cohort, such as from the Carotene and Retinol Efficacy Trial (CARET) study [170] and the Prostate, Lung, Colorectal, Ovarian (PLCO) Cancer Screening Trial [171], could provide insight into the potential of such antibodies for early cancer detection. However, a large prospective study would ultimately be required. Before any such study would be possible, stronger data justifying the feasibility of autoantibody markers for early SCLC detection would be needed. To this end, further analysis of the SCLC-prone mouse model system [48,131] could provide important data on the potential sensitivity and specificity of antigen panels.

While early detection of SCLC would be the holy grail, a much simpler application of SCLC-associated autoantibodies is the establishment of a diagnosis. If the timing of autoantibody generation does *not *precede cancer development, or does not precede it by sufficient time, these antibodies would still be useful tools to establish or confirm a SCLC diagnosis. In PNS patients, detected autoantibodies often trigger a tumor search. One could envision that an antigen panel to confirm suspected SCLC could be established. Retrospective studies of archival human serum and plasma aimed at exploring SCLC-associated antibodies for early detection will provide data that would also be very useful to determine utility for diagnosis. Ultimately, however, a prospective study in which serum and plasma from suspected SCLC are tested and correlated with the confirmed clinical diagnosis, would be required.

### Survival and prognostic benefits of a SCLC-associated immune response

How could SCLC-associated autoantibodies affect survival? For one, in patients exhibiting neurological symptoms, a diagnosis of SCLC-related autoimmunity will prompt a tumor search, which would allow the cancer to be detected at an earlier time point when intervention might be more effective. The fact that autoantibodies can precede the diagnosis of the tumor [172,173] suggests the cancer can still be very small, and it opens a window for improving the overall outcome in these patients, perhaps by beginning antitumor treatment before metastasis occurs.

Secondly, the immune response may itself be protective. A number of studies suggest that patients with SCLC-related autoimmune disease have a better prognosis than patients with histologically identical tumors without a paraneoplastic disorder [139,174-176]. The fact that some PEM/SN patients have small or virtually undetectable SCLC lesions that are only identifiable at autopsy supports this observation [42]. Furthermore, it has been reported that in SCLC patients *without *paraneoplastic disease, low titers of anti-Hu autoantibodies are correlated with comparatively indolent tumor growth relative to antibody-negative patients [42,88]. The presence of other SCLC-associated autoantibodies (VGCCs, CV2/CRMP5) in patients has been correlated to slower tumor growth, complete response to therapy, and longer survival in some studies [32,41,42,52,88,137-142]. Other studies, however, have found no survival benefit in correlation with SCLC-associated autoantibodies [28,31,50,88,143,144]. This discrepancy may be due to sample size, treatment protocols, secondary endpoints used to measure survival, the nature of the antibodies, the presence of clinical features, and whether or not the clinical status of the patient is known at the close of the study.

Abnormal or ectopic expression of antigens by a non-neuronal cell type could activate immuno-competent cells that promote an inflammatory response. The inflammatory response can trigger apoptosis, which could then initiate an antitumor immune response [22,108]. Apoptosis of tumor cells can expose intracellular antigens, mediating an immune response to self-antigens that are expressed both in the tumor and the nervous system [177-179]. Furthermore, tumor cells can be phagocytosed by dendritic cells that further activate antigen-specific CD4+ and CD8+ T cells and B cells through normal immunologic processes. These antibody-producing B cells could act in concert along with cytotoxic T cells specific for the tumor antigen and induce cytotoxicity, potentially retarding tumor growth. The antibodies could trigger complement-mediated cytotoxicity [180] or antibody-dependent cell-mediated cytotoxicity [181]. Furthermore, the antibodies may bind to their target and interfere with the function of the antigen. This might disrupt tumor progression if the antigen is necessary for tumor growth. The latter may provide an explanation for how tumors in some antibody-positive SCLC patients are smaller or slower growing. Definitive evidence of this theory is lacking, however. The nature of the immuno-response in SCLC requires further characterization to determine the protective nature of antibodies and T cells. Again, the SCLC-prone mouse model discussed in Section 5 would be a useful resource to study this phenomenon.

### SCLC autoantigens as targets for anti-cancer therapy and imaging

The development of SCLC vaccines presents a formidable challenge because an immune response against antigens expressed both in the tumor and nervous system may cause neurotoxicity. The main objective in treating a patient with SCLC-related PNS is to cure the underlying cancer and to improve or stabilize the neurological dysfunction [140]. Interestingly, in mice that were implanted with neuroblastoma, immunization with DNA encoding HuD has been shown to retard tumor growth [110,111], and the mice did not develop neurologic abnormalities in this study nor in a study where mice were immunized with recombinant purified HuD antigen [111]. The SCLC-prone mouse model [131] can be utilized in a similar manner to determine if immunization with various SCLC-associated autoantigens would protect against the development of the SCLC tumor; however, neurotoxicity is a potential side effect and would need to be closely monitored.

A very different situation arises if the antigens triggering the immune responses are mutated or post-translationally modified in the tumor compared to the native nervous system. In that case, a strategy to specifically target the modification would be an option. However, when using immunizations aimed at inducing a cancer-specific response to a modified protein, a unique tumor-specific epitope may not preclude a destructive autoimmune response; reactivity might expand through epitope spreading. For any immunotherapy strategy, finding the right balance between attacking the tumor and avoiding neurotoxicity is imperative. A more promising avenue may be to develop molecules tailored to recognize the antigen using *in vitro *evolution in small scaffolds, like knottin peptides [182]. Such "magic bullets" could be targeted toward any cancer-specific modification to deliver molecules for imaging or treatment. Visualization of small tumors is critical to establish the precise location to perform successful resection. Most current imaging modalities are not specific for cancer, although metabolism-based markers show higher signals in cancer cells based on their increased metabolic activity. Imaging agents specifically targeted to cancer cells can be powerful aids in cancer detection and could increase our ability to detect small SCLC lesions.

The potential to use the immune system or targeted molecules to attack the tumor underlines the importance of obtaining a detailed mechanistic understanding of SCLC-associated autoimmunity. Until the nature of the antigen and the immune response are clarified, it will be very difficult to devise fruitful applications. The biggest challenge may be that different SCLC-associated immune responses may occur through distinct mechanisms.

## Conclusions

Identifying SCLC-associated autoantigens that may elicit an antibody-based immune response may yield new methods for SCLC detection, screening, treatment, and imaging. Antibody responses against a wide variety of antigens are seen in SCLC patients. How common these antibodies are in patients and what the background response is in healthy individuals varies by antigen. The exact mechanism whereby these antibodies develop remains to be elucidated. While the examination of antibodies for a single autoantigen as a diagnostic or screening tool for SCLC would not be sufficiently sensitive given the fact that only a fraction of SCLC patients shows a response to any given antigen, in theory, a *panel *of antigens might be used to detect antibodies in the blood of those at most risk for SCLC. It is unclear whether such a panel could provide high enough sensitivity and specificity and whether the antibodies arise with enough lead time to allow intervention with an appreciable impact on survival. Effective intervention would only be possible if the tumor could be resected before it metastasizes or if improved treatments become available. Establishing the timing of an autoimmune response relative to cancer development is therefore of great importance. The identification of the autoimmune trigger (if it is distinct from the wildtype neuron-specific protein) in the different SCLC-associated PNS is crucial. Any identified epitopes might be of use as therapeutic targets, for example using tailored antibodies or synthetic molecules. A recently developed SCLC mouse model that has been shown to develop an anti-Hu response provides powerful new tool for investigation in this important field, and could provide insight into many of the questions raised above.

## Competing interests

The authors declare that they have no competing interests.

## Authors' contributions

MK and IALO reviewed the literature, and drafted, edited, and finalized the manuscript.
